# Genome-wide study of longitudinal brain imaging measures of multiple sclerosis progression across six clinical trials

**DOI:** 10.1038/s41598-023-41099-0

**Published:** 2023-08-31

**Authors:** Stephanie J. Loomis, Nilanjana Sadhu, Elizabeth Fisher, Arie R. Gafson, Yunfeng Huang, Chengran Yang, Emily E. Hughes, Eric Marshall, Ann Herman, Sally John, Heiko Runz, Xiaoming Jia, Tushar Bhangale, Paola G. Bronson

**Affiliations:** 1 Biogen, Cambridge, MA USA; 2 Genentech, South San Francisco, CA USA

**Keywords:** Genome-wide association studies, Multiple sclerosis

## Abstract

While the genetics of MS risk susceptibility are well-described, and recent progress has been made on the genetics of disease severity, the genetics of disease progression remain elusive. We therefore investigated the genetic determinants of MS progression on longitudinal brain MRI: change in brain volume (BV) and change in T2 lesion volume (T2LV), reflecting progressive tissue loss and increasing disease burden, respectively. We performed genome-wide association studies of change in BV (N = 3401) and change in T2LV (N = 3513) across six randomized clinical trials from Biogen and Roche/Genentech: ADVANCE, ASCEND, DECIDE, OPERA I & II, and ORATORIO. Analyses were adjusted for randomized treatment arm, age, sex, and ancestry. Results were pooled in a meta-analysis, and were evaluated for enrichment of MS risk variants. Variant colocalization and cell-specific expression analyses were performed using published cohorts. The strongest peaks were in *PTPRD* (rs77321193-C/A, p = 3.9 × 10^–7^) for BV change, and *NEDD4L* (rs11398377-GC/G, p = 9.3 × 10^–8^) for T2LV change. Evidence of colocalization was observed for *NEDD4L*, and both genes showed increased expression in neuronal and/or glial populations. No association between MS risk variants and MRI outcomes was observed. In this unique, precompetitive industry partnership, we report putative regions of interest in the neurodevelopmental gene *PTPRD*, and the ubiquitin ligase gene *NEDD4L*. These findings are distinct from known MS risk genetics, indicating an added role for genetic progression analyses and informing drug discovery.

## Introduction

Multiple sclerosis (MS) is an immune-mediated disorder of the central nervous system (CNS) characterized by clinical relapses with associated acute focal brain inflammation, and progressive worsening of disability with accompanying neurodegeneration. Disease outcomes are highly variable in persons with MS (PwMS)^1^ and injury contributing to disease occurs across the disease course, whether in the presence or absence of relapses^2^. While current MS disease-modifying therapies (DMTs) effectively suppress acute disease activity, development of novel treatments that effectively reduce disability progression remains an unmet need.

There is a strong genetic component for MS risk, with > 200 confirmed genome-wide significant variants which, along with variants in the extended major histocompatibility complex (MHC) region, account for approximately 38.2% of the overall heritability of MS^3^. These loci primarily affect the immune system, supporting the autoimmune hypothesis of MS pathogenesis and the use of available immune-modulating DMTs. Recently, a large MS study highlighted the importance of CNS resilience for severity^4^, and furthermore, did not observe overlap between severity and susceptibility loci. However, little is known about the genetics of MS progression, which may also differ from genetics of MS susceptibility. The paucity of progression genetics knowledge could possibly be due to the lack of meaningful longitudinal data at scale. Previous studies have used cross-sectional imaging traits and non-linear MS disability and severity scores to investigate MS severity^5–8^. Additionally, measuring MS disease progression using clinical outcomes is challenging. The Expanded Disability Severity Score (EDSS) is commonly used to measure worsening, but only partially captures underlying insidious injury^9^. Similarly, clinical relapses in the early stages of MS do not predict long-term outcomes in natural history studies^10^.

Therefore, we leveraged longitudinal imaging data from a unique dataset of randomized controlled trials (RCTs) conducted by Biogen and Roche/Genentech in a precompetitive partnership to examine the genetics of MS progression. We investigated genetic correlates of two longitudinal, quantitative brain MRI outcomes: change in brain volume (BV), and change in T2 lesion volume (T2LV). BV change measures the overall impact of MS injury, including demyelination and neurodegeneration^11^. The rate of brain atrophy over time is a quantitative measure that correlates with worsening clinical disability^12,13^. T2LV increases with accumulating injury and early measures of T2LV may be predictive of long-term disability^14,15^. A deeper understanding of the genetic factors that impact the rate of MS progression could reveal novel candidate targets and biomarkers relevant to MS progressive biology.

## Materials and methods

### Standard protocol approvals, registrations, and patient consents

We performed GWAS of BV change (N = 3401) and T2LV change (N = 3513) across six RCTs: ADVANCE (trial registry number: NCT00906399), ASCEND (trial registry number: NCT01416181), DECIDE (trial registry number: NCT01064401), OPERA I & II (trial registry number: NCT01247324, NCT01412333), and ORATORIO (trial registry number: NCT01194570) (eTables 1, 2). All participants provided written informed consent that covered the scope of this research. Ethical approval was provided as per the original RCTs. This work adheres to the Declaration of Helsinki.

### Image acquisition and trait estimation

T1-weighted, proton density-weighted and T2-weighted brain MRIs were collected in accordance with standardized imaging protocols for each trial. All trials used the same centralized MRI reading center (NeuroRx, Montreal, Canada).

BV change was measured between week 24 and the last time point available for each participant using the automated SIENA (Structural Image Evaluation, using Normalization, of Atrophy) method^16^. We used week 24 instead of baseline to account for potential rapid volume changes related to resolution of acute inflammation following treatment initiation (also known as pseudoatrophy)^12^. For placebo arm patients in ADVANCE, BV change was calculated from baseline to week 48 (when participants switched to active treatment).

To calculate T2LV, T2 lesions were segmented using a fully-automated method, followed by visual verification and adjustments, if needed. T2LV change was then estimated as the change in total T2LV between baseline and last time point available for each participant. For the placebo arm in ADVANCE, we used week 48 instead of the last time point to be consistent with the BV change estimation.

Within each RCT, changes in BV and T2LV were annualized to account for varying lengths of follow-up time between the trials. This was accomplished by dividing each individual change by the number of years between the first and last time point used in estimation. Annualized traits were transformed using rank-based inverse normal transformation to mitigate departures from normality (eFigures 1, 2).

### Genetic data generation and quality control (QC)

#### ADVANCE, ASCEND and DECIDE (Biogen trials)

DNA was extracted from blood and genotyped with the Affymetrix UK Biobank Axiom array by Thermo Fisher Scientific (Santa Clara, CA) in two batches: ASCEND and DECIDE (2017), and ADVANCE (2018). QC (PLINK v1.9) and imputation (Michigan Imputation server) was run on the batches separately (hg19). We excluded single nucleotide polymorphisms (SNPs) with missingness > 1%, minor allele frequency (MAF) < 0.01, and Hardy–Weinberg equilibrium (HWE) p < 1 × 10^–50^. As these are case-only cohorts, we applied a less stringent HWE threshold to avoid the inadvertent exclusion of disease-associated variants. We excluded samples with missingness > 2%, sex discrepancies (females: F < 0.2, males: F > 0.8), excess heterozygosity (> 6 standard deviations from the mean), and relatedness. Independent SNPs (PLINK option --indep-pairwise 50 5 0.5) were used to estimate sex, heterozygosity, relatedness, and ancestry.

Identity by descent (IBD) analysis was used to identify related individuals. Pairs of individuals with k0 > 0.4 were considered related, and one member from each pair was selected randomly for exclusion. We estimated ancestry separately in each RCT, and used principal component analysis (PCA; SmartPCA, Eigensoft v7.2.1) to identify outliers and calculate PCs. We excluded individuals who were 6 standard deviations from the top 10 PCs with a maximum of 10 outlier removal iterations (default). We estimated Tracy-Widom (TW)^17^ statistics to determine the number of PCs to include as covariates, which were defined as PCs with TW p < 0.05 that accounted for ≥ 1% of variance (calculated by subtracting the largest eigenvalue with p ≥ 0.05, from the sum of the eigenvalues with p < 0.05, and then estimating relative contributing variance).

Genotypes were imputed to 1000G phase3v5 (phasing: ShapeIT v2.r790). After imputation, we excluded SNPs with R^2^ < 0.30, MAF < 0.01 or in the pseudo-autosomal region of chromosome X. SNPs and samples at each QC step are tabulated in eTable 3.

#### OPERA I, OPERA II, and ORATORIO (Roche trials)

DNA was extracted from blood and whole-genome sequenced (WGS, mean read depth 30x) (TruSeq Nano library prep, Illumina HiSeq) to generate 150 bp paired-end reads (Illumina, Inc [Foster City, CA]). Burrows-Wheeler Aligner (BWA) was used to map reads (hg38). Resulting alignments (bam files) were analyzed using GATK for base quality score recalibration (BQSR), indel realignment, duplicate removal, SNP/INDEL discovery and joint genotyping across samples according to GATK Best Practices^18^. Sites that did not pass GATK variant quality score recalibration (VQSR) filter were removed. Genotypes with quality score (GQ) < 20 were set to missing, followed by removal of sites with missing rate > 10%. To further improve the coverage of common variants, genotype imputation was performed using a haplotype reference panel of 55,929 individuals constructed using 27,166 individuals from Haplotype Reference Consortium, 2548 samples from 1000 Genomes project and 26,215 samples sequenced at Genentech. Imputation was performed using Beagle version 5.2 and sites with imputation score < 0.3 or HWE p < 5 × 1^–10^ or MAF < 1% were removed.

Ancestry was estimated using ADMIXTURE (supervised mode; reference 1000G phase3). Samples with European ancestry coefficient < 0.7 were excluded. Analyses for heterozygosity outlier detection, relatedness (IBD, where pairs of individuals with k0 < 0.4 were considered related), TW statistic, and PCA were performed similarly to Biogen.

### GWAS statistical analysis

We used linear regression models to test the association between SNPs with BV change and T2LV change (additive model), controlling for randomized treatment arm, age, sex, and genetic ancestry (3 PCs in ADVANCE and DECIDE, 4 PCs in ASCEND, and 11 PCs in OPERA I & II and ORATORIO). Biogen analyses were performed using imputed dosages (PLINK v2.0 option --glm --maf 0.01). Genentech analyses were performed using WGS (PLINK v1.9 option --linear --maf 0.01). Summary statistics from Roche RCTs were mapped from hg38 to hg19 using liftOver in Hail.

Summary statistics from RCTs were meta-analyzed using a fixed-effects model (PLINK v1.9). Regional plots were generated with LocusZoom v1.4 (1000G phase3 EUR).

To increase power beyond single variant analysis, we used MAGMA^19^ to evaluate associations on the gene level using the meta-analyzed summary statistics. We used the most recent version of MAGMA (1.10) with the reference data provided by the authors of MAGMA, which was generated from Phase 3 of the 1000 Genomes Project. We used a Bonferroni correction to determine the significance threshold (0.05/17,837 genes = 2.6 × 10^–6^).

### Colocalization analysis

To better understand which genes that our top hits for BV change and T2LV change may impact, we performed colocalization (adapted from coloc package) with human expression data from 48 GTEx tissues (https://gtexportal.org/) and the Database of Immune Cell eQTLs (https://dice-database.org/). We also examined evidence for shared causal variants between selected MS risk loci and our top hits (p < 1 × 10^–6^). For each analysis, we included all SNPs within a 250 kb window (500 kb region) of the query study SNP (static default prior probabilities: p1 = p2 = 1 × 10^–4^; p12 = 1 × 10^–5^).

### CNS cell-type specific expression

To visualize single-nucleus gene expression of putative genes identified through GWAS meta-analysis by CNS cell type, we used *CellxGene VIP* (https://cellxgenevip-ms.bxgenomics.com/). This is based on brain tissue from 12 MS patients^20^.

### Comparison with MS risk SNPs

We examined all autosomal MS risk GWAS loci^3^, including peak variants in the MHC, for potential association with change in BV and T2LV. We selected available overlapping variants from WGS and genotype data, and report association statistics for variants whose effects on BV change and T2LV change were consistent across the six RCTs (heterogeneity I^2^ < 40). Additionally, we generated a polygenic risk score (PRS) using the non-MHC variants and weights from the IMSGC MS risk discovery GWAS, and tested the score for association with change in T2LV and change in BV^3^.

## Results

GWAS meta-analysis included N = 3401 participants for BV change and N = 3513 participants for T2LV change from RCTs in relapsing remitting MS (RRMS) [ADVANCE (N = 540; 505, for BV and T2LV change data, respectively), DECIDE (N = 821; 886), OPERA I (N = 581; 581), OPERA II (N = 577; 577)], secondary progressive MS (SPMS) (ASCEND; N = 353; 435), and primary progressive MS (PPMS) (ORATORIO, N = 529; 529) (Table 1). Participants were mainly female (51–74%), mean age was higher in the PMS RCTs (45–47 vs 36–38 in RRMS), and mean study duration was ~ 1.8 years. In total, we meta-analyzed 10,382,375 and 10,608,740 SNPs for association with BV change and T2LV change, respectively. The genomic inflation factor did not show evidence of bias from ancestry in either GWAS (λ_BV_ = 1.008, λ_T2LV_ = 1.019) (eFigure 3).

**Table 1:** Sample characteristics for study population.

	Biogen			Genentech/Roche		
Trial	ADVANCE	ASCEND	DECIDE	OPERA1	OPERA2	ORATORIO
MS stage	RRMS	SPMS	RRMS	RRMS	RRMS	PPMS
Active arm	Pegylated IFNβ	Natalizumab	Daclizumab	Ocrelizumab	Ocrelizumab	Ocrelizumab
Control arm	Placebo	Placebo	IFNβ	IFNβ	IFNβ	Placebo
**Change in brain volume GWAS**						
Study duration in years, mean	1.2	1.4	1.7	1.3	1.3	1.5
Active arm, N	347^a^	175	423	297	284	354
Control arm, N	193	178	398	284	293	175
Total, N	540	353	821	581	577	529
Baseline characteristics						
Age in years, mean (SD)	37 (9.7)	47 (7.6)	36 (9.5)	38 (9.1)	38 (9.1)	45 (7.7)
Female, %	73	62	66	66	64	51
Normalized BV in cm^3^, mean (SD)	1580 (94)	1426 (82)	1502 (89)	1492 (86)	1494 (92)	1460 (85)
**Change in T2 lesion volume GWAS**						
Study duration in years, mean	1.6	1.8	1.8	1.7	1.7	2.0
Active arm, N	354^b^	214	454	297	284	354
Control arm, N	151	221	432	284	293	175
Total, N	505	435	886	581	577	529
Baseline characteristics						
Age in years, mean (SD)	37 (9.7)	47 (7.5)	36 (9.3)	38 (9.1)	38 (9.1)	45 (7.7)
Female, %	74	62	66	66	64	51
T2LV in cm^3^, mean (SD)	10.1 (11.7)	17.4 (17.3)	9.4 (11.1)	9.9 (12.3)	10.9 (14.1)	11.6 (13.7)

BV, brain volume; PPMS, primary progressive multiple sclerosis; RRMS, relapsing remitting multiple sclerosis; SD, standard deviation; SPMS, secondary progressive multiple sclerosis; T2LV, T2 lesion volume.

^a^Biweekly: 178, monthly: 169.

^b^Biweekly: 178, monthly: 176.

**Figure 1:**
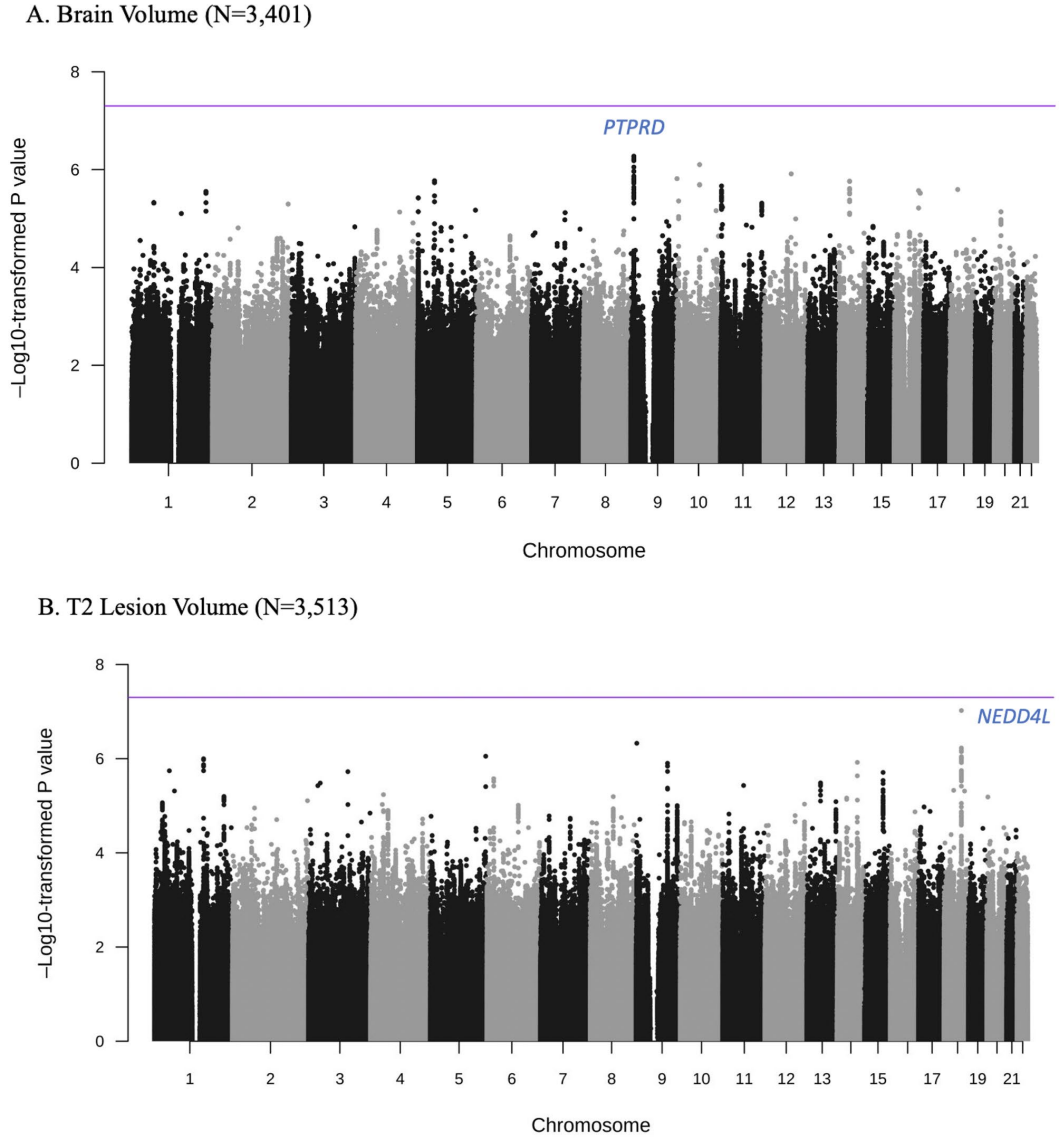
Genome-wide association plots from meta-analysis of change in brain volume (BV) and T2 lesion volume (T2LV) GWAS studies. Manhattan plots with negative log_10_ P-values on y-axis, and chromosome position (hg19) on x-axis. Each point corresponds to a single SNP analyzed in the GWAS: (**A**) BV change (N = 3401); (**B**) T2LV change (N = 3513). Horizontal purple line indicates genome-wide significance threshold (p = 5 × 10^–8^). Peak SNPs labeled with closest gene(s). BV, brain volume; GWAS, genome-wide association study; *NEDD4L*, neural precursor cell-expressed developmentally down-regulated 4-like; *PTPRD*, protein tyrosine phosphatase receptor type D; SNP, single nucleotide polymorphism; T2LV, T2 lesion volume.

**Figure 2:**
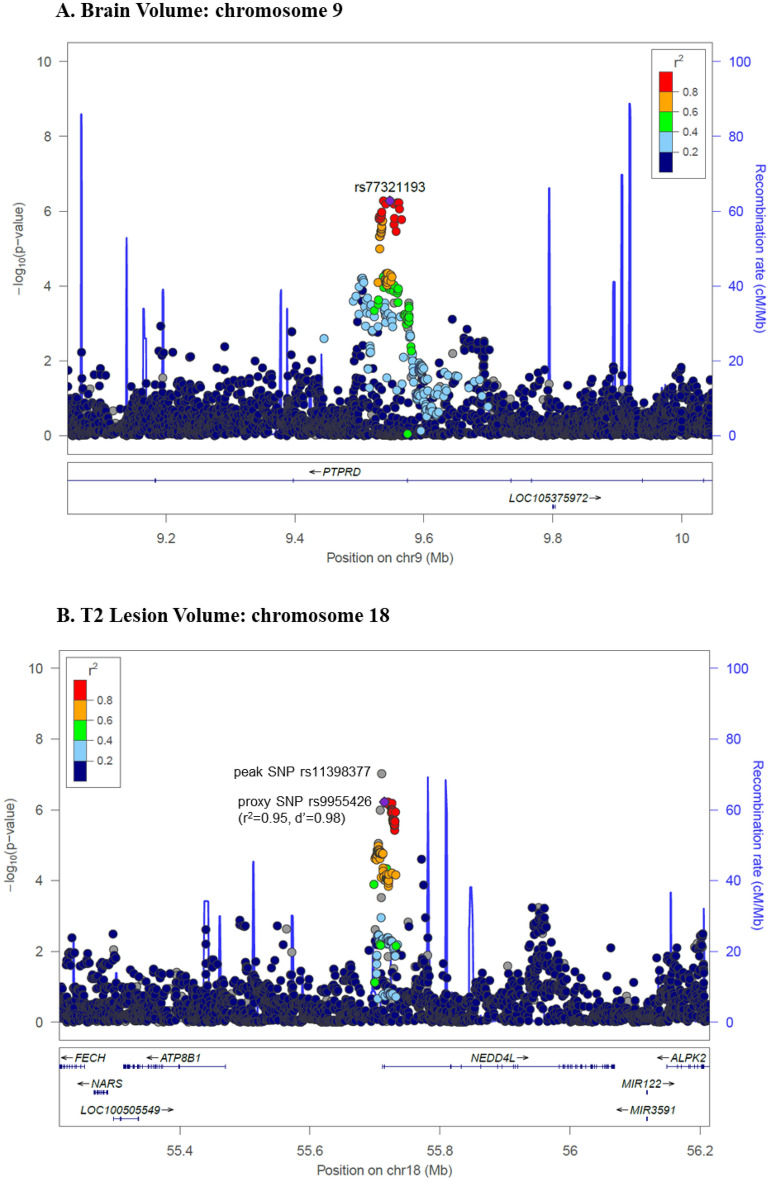
Regional association plots of peak SNPs from meta-analysis of change in brain volume (BV) and T2 lesion volume (T2LV) GWAS studies. Regional association plots with negative log_10_ P-values on primary y-axis, genetic recombination rate on secondary y-axis, and chromosome position (hg19) on x-axis. Each point corresponds to a single SNP analyzed in the GWAS. The correlation between the peak SNP (or proxy SNP) and the other SNPs in the region is reflected by the linkage disequilibrium (*r*^*2*^) estimate, depicted in blue for least correlation (*r*^*2*^ < 0.2) to red for most correlation (*r*^*2*^ > 0.8). The plots were created using LocusZoom (http://locuszoom.sph.umich.edu/), and depict the association of: (**A**) BV change with intronic SNP rs77321193 in *PTPRD* on chromosome 9; (**B**) T2LV change with regulatory SNP rs11398377 near *NEDD4L* on chromosome 18. Proxy SNP rs9955426 was used for plot B because rs11398377 was not present in the reference panel (1000G EUR). 1000G EUR, 1000 Genomes European; BV, brain volume; GWAS, genome-wide association study; *NEDD4L*, neural precursor cell-expressed developmentally down-regulated 4-like; *PTPRD*, protein tyrosine phosphatase receptor type D; SNP, single nucleotide polymorphism; T2LV, T2 lesion volume.

**Figure 3:**
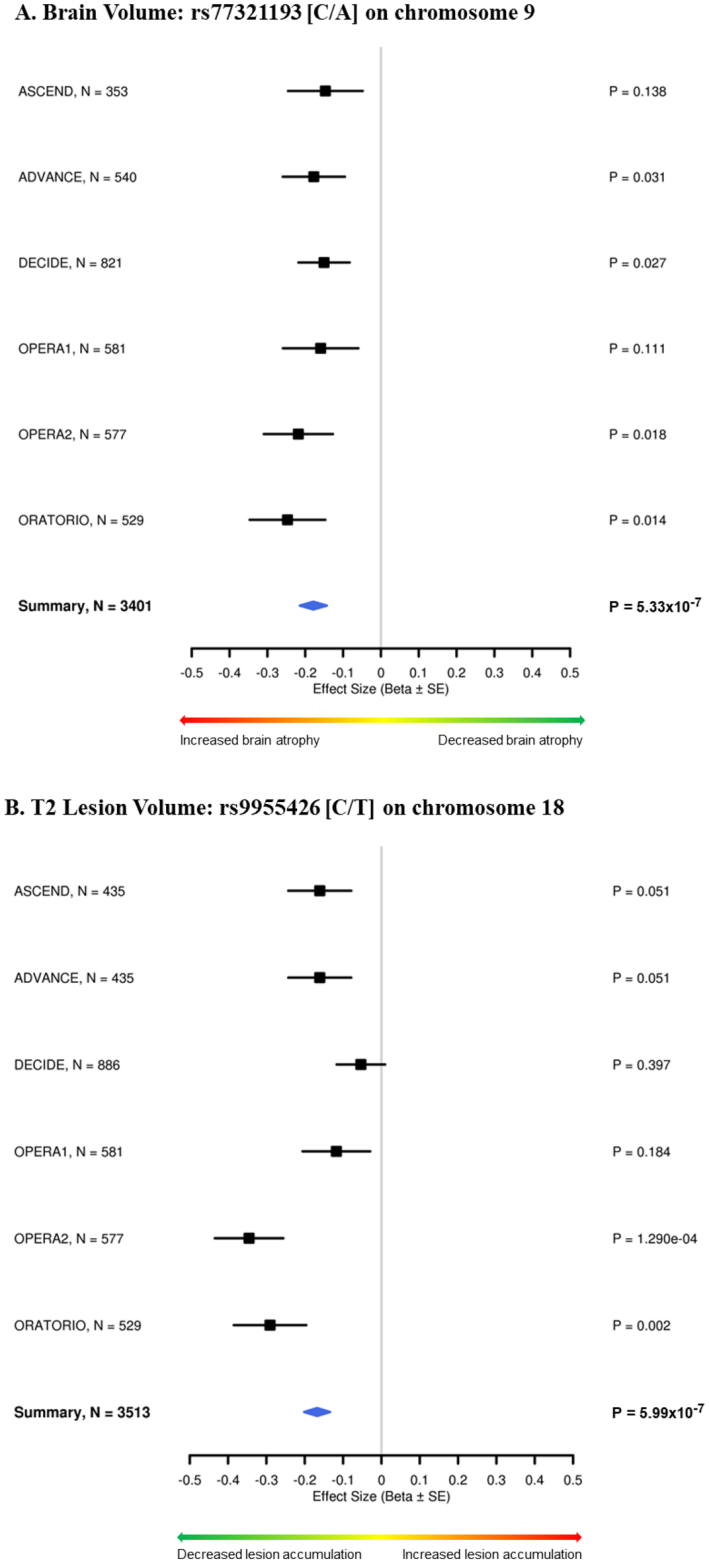
Forest plots of peak SNPs from meta-analysis of change in brain volume (BV) and T2 lesion volume (T2LV) GWAS studies. Forest plots depicting trial-wise (black squares) and meta-analyzed (summary in blue diamond) association of: (**A**) peak variant rs77321193 on chromosome 9 with BV change (effect allele: C, heterogeneity I^2^ < 0.01, p = 0.95); (**B**) Proxy SNP (for peak variant) rs9955426 on chromosome 18 with T2LV change (effect allele: C, heterogeneity I^2^ = 46, p = 0.10). Effect sizes are reported as Beta ± SE on x-axis, sample sizes are reported as N along with trial names, and strength of associations are reported as P-values (P). Direction of association is indicated on color scale at the bottom of each plot. BV, brain volume; GWAS, genome-wide association study; *NEDD4L*, neural precursor cell-expressed developmentally down-regulated 4-like; *PTPRD*, protein tyrosine phosphatase receptor type D; SNP, single nucleotide polymorphism; T2LV, T2 lesion volume.

**Figure 4:**
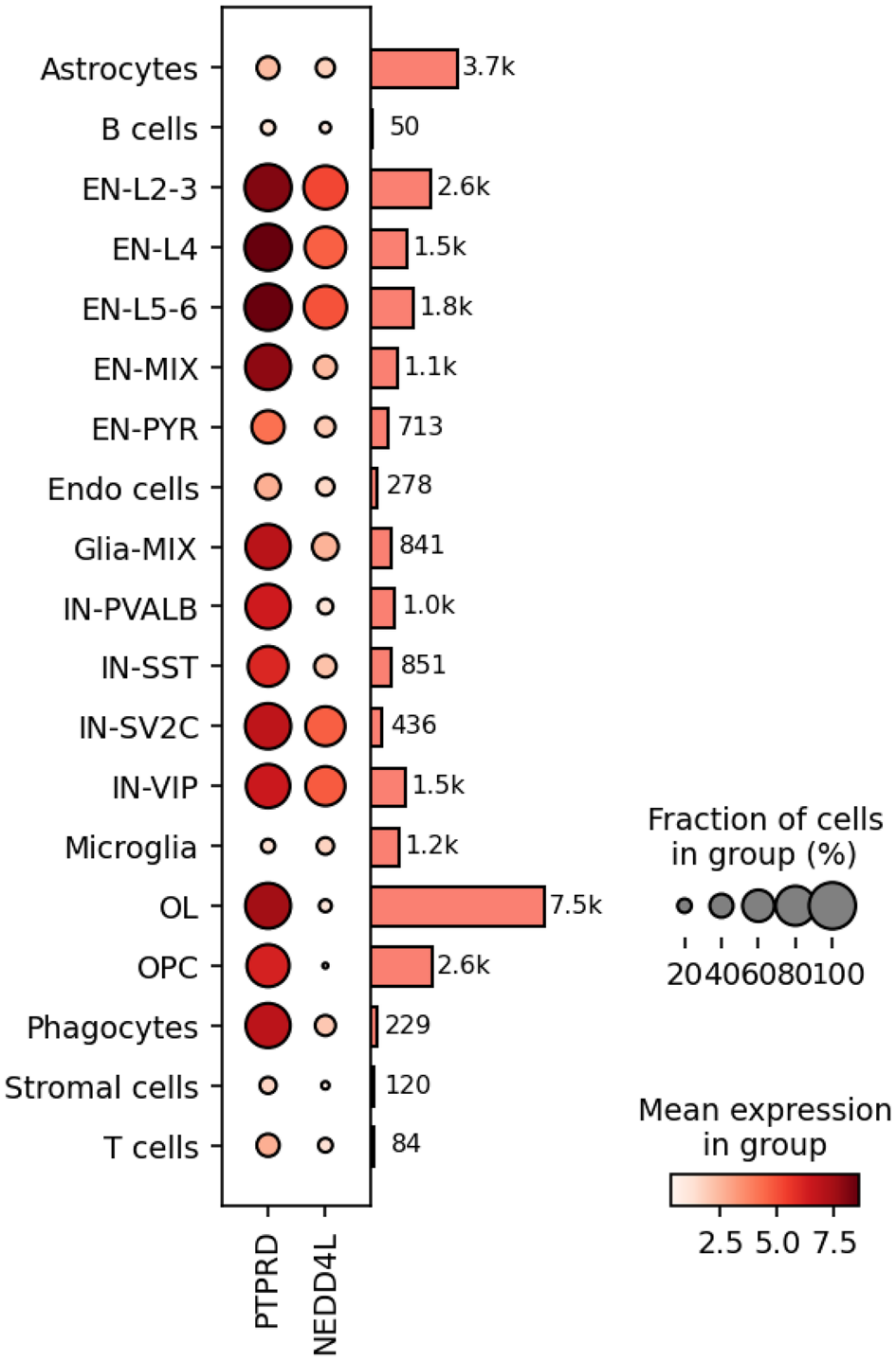
Dot plot of CNS cell-type specific expression of *PTPRD*, *NEDD4L* in MS brain tissue. CNS, central nervous system; EN, excitatory neuron; IN, inter-neuron; L2-3, upper layer cortical projection; L4, layer 4 cortical projection; L5-6, deep layer cortical projection; MIX, mixed; MS, multiple sclerosis; OL, oligodendrocyte; OPC, oligodendrocyte progenitor cell; PYR, pyramidal cell; PVALB, parvalbumin; SST, somatostatin; SV2C, synaptic vesicle glycoprotein 2C; VIP, vasoactive intestinal peptide.

The most significant association with BV change was an intronic SNP in *PTPRD* (top SNP: rs77321193-C/A, p = 5.3 × 10^–7^), which showed no evidence for heterogeneity across the trials (heterogeneity I^2^ = 0, heterogeneity p = 0.96) (Fig. 1A, Table 2). The minor allele (C; MAF = 0.18) of rs77321193 was associated with a greater reduction in BV over time. Multiple SNPs in this region showed association with BV change with similar effect sizes and no evidence for heterogeneity in the meta-analysis (eTable 4, Figs. 2A, 3A). We did not observe colocalization between the *PTPRD* peak and expression of *PTPRD* in the datasets we evaluated. However, analysis of cell-type specific gene expression in MS brain (Fig. 4) revealed that *PTPRD* was expressed in neurons, oligodendrocytes and oligodendrocyte precursor cells. The gene-based tests did not reveal significant association with *PTPRD* (p = 0.066), or other genes (top hit: *TSPAN8*, p = 8.08 × 10^–5^) (eTable 5).

**Table 2:** Peak SNPs from meta-analysis of change in brain volume (BV) GWAS study.

SNP	CHR	BP	A1/A2	A1 Frequency^a^	Beta (SE)	P-value	Nearest gene	Context
rs10491610	9	9537211	T/G	0.18	− 0.18 (0.04)	5.43 × 10^–7^	*PTPRD*	Intronic
rs17772815	9	9540766	C/G	0.18	− 0.18 (0.04)	6.57 × 10^–7^		Intronic
rs77321193	9	9547291	C/A	0.18	− 0.18 (0.04)	5.33 × 10^–7^		Intronic
rs736043	9	9553360	C/T	0.18	− 0.18 (0.04)	6.39 × 10^–7^		Intronic
rs76647005	9	9558543	C/T	0.18	− 0.18 (0.04)	5.93 × 10^–7^		Intronic
rs77127788	9	9561124	A/G	0.18	− 0.18 (0.04)	6.04 × 10^–7^		Intronic
rs6477402	9	9562254	C/T	0.18	− 0.18 (0.04)	8.89 × 10^–7^		Intronic
rs137996531	10	71059147	A/G	0.04	0.46 (0.09)	7.90 × 10^–7^	*HK1*	Intronic

A1, Allele 1 (effect allele); A2, Allele 2; BV, brain volume; GWAS, genome-wide association study; *PTPRD*, protein tyrosine phosphatase receptor type; SNP, single nucleotide polymorphism.

Fixed-effect meta-analysis results with p < 1 × 10^–6^ and low heterogeneity (Cochrane’s Q p-value > 0.05 for all SNPs).

^a^Allele frequency in the 1000G phase3 European cohort.

The strongest association with T2LV change was in a regulatory region near *NEDD4L* (top SNP: rs11398377-GC/G, p = 9.5 × 10^–8^), which was consistent across the trials (heterogeneity I^2^ = 51, heterogeneity p = 0.07) (Fig. 1B, Table 3). The C deletion in rs11398377 (MAF = 0.16) was associated with greater T2LV accumulation over time. Several SNPs in this region associated with T2LV change had similar effect sizes and did not show significant heterogeneity in the meta-analysis (eTable 6, Figs. 2B, 3B). Interestingly, we found evidence for colocalization between the T2LV change peak (rs11398377) and *NEDD4L* expression in whole blood (colocalization pp4 = 0.78), suggestive of a common causal variant between the traits. Moreover, analysis of cell-specific gene expression in the MS brain showed that *NEDD4L* is expressed mainly in neurons, including inter-neurons and excitatory neurons (Fig. 4). Gene-based tests did not show significant associations with change in T2LV (top hit: *WDR34*, p = 2.0 × 10^–5^), however *NEDD4L* was among the top hits (p = 6.94 × 10^–5^) (eTable 7).

**Table 3:** Peak SNPs from meta-analysis of change in T2LV GWAS study.

SNP	CHR	BP	A1/A2	A1 Frequency^a^	Beta (SE)	P-value	Nearest gene	Context
rs11398377	18	55710518	GC/G-	0.84	− 0.18 (0.03)	9.52 × 10^–8^	*NEDD4L*	2 kb upstream
rs9955426	18	55714622	C/T	0.84	− 0.17 (0.03)	5.99 × 10^–7^		5′ UTR
rs8093023	18	55720092	A/G	0.84	− 0.17 (0.03)	5.94 × 10^–7^		Intronic
rs8097163	18	55720610	A/G	0.84	− 0.17 (0.03)	7.11 × 10^–7^		Intronic
rs6566928	18	55725195	C/T	0.84	− 0.17 (0.03)	9.07 × 10^–7^		Intronic
rs6566931	18	55725783	A/G	0.84	− 0.17 (0.03)	9.51 × 10^–7^		Intronic
rs5005280	18	55725820	G/A	0.84	− 0.17 (0.03)	9.45 × 10^–7^		Intronic
rs1942562	18	55725858	C/T	0.84	− 0.17 (0.03)	9.57 × 10^–7^		Intronic
rs4371241	18	55725925	A/C	0.84	− 0.17 (0.03)	9.68 × 10^–7^		Intronic
rs1942563	18	55725956	C/G	0.84	− 0.17 (0.03)	9.73 × 10^–7^		Intronic
rs4461163	18	55726087	T/G	0.84	− 0.17 (0.03)	6.64 × 10^–7^		Intronic
rs1942564	18	55726105	A/G	0.84	− 0.17 (0.03)	9.87 × 10^–7^		Intronic
rs1942565	18	55726152	C/T	0.84	− 0.17 (0.03)	9.88 × 10^–7^		Intronic
rs4375742	18	55726189	T/C	0.84	− 0.17 (0.03)	9.86 × 10^–7^		Intronic
rs12237424	9	2442058	A/G	0.08	− 0.23 (0.05)	4.71 × 10^–7^	*LOC101930053*	Intronic
rs145565113	5	176921677	A/G	0.03	− 0.49 (0.10)	8.88 × 10^–7^	*PDLIM7*	Intronic

A1, Allele 1 (effect allele); A2, Allele 2; GWAS, genome-wide association study; *NEDD4L*, neural precursor cell-expressed developmentally down-regulated 4-like; SNP, single nucleotide polymorphism; T2LV, T2 lesion volume.

Fixed-effect meta-analysis results with p < 1 × 10^–6^ and low heterogeneity (Cochrane’s Q p-value > 0.05 for all SNPs).

^a^Allele frequency in the 1000G phase3 European cohort.

Comparison with the known MS risk GWAS^3^ SNPs (197 non-MHC and autosomal; 161 and 162 SNPs met our inclusion criteria for comparison with BV change and T2LV change meta-analyses results, respectively) did not show evidence of overlap with BV change nor T2LV change GWAS hits (eTables 8, 9). We did not observe any significant associations (p < 1 × 10^–6^) between MS susceptibility loci with BV change or T2LV change, and found no overlap with MS risk loci in the MHC region^3^. Conversely, neither the BV change peak in *PTPRD* nor the T2LV change peak in *NEDD4L* were associated with MS risk^3^, and no putative BV change or T2LV change peaks (p < 1 × 10^–6^) colocalized with MS risk. Additionally, the MS risk PRS was not significantly associated with change in BV (p = 0.54) nor with change in T2LV (p = 0.89).

## Discussion

To our knowledge, this is the first GWAS meta-analysis of longitudinal MRI measures across multiple RCTs to investigate the genetics of MS progression. We achieved this by leveraging a precompetitive industry partnership aimed at utilizing well-characterized MS cohorts to interrogate the genetics of disease progression in MS.

The top BV change SNP occurs in an intron of *PTPRD*, a protein tyrosine phosphatase receptor involved in cellular signaling, growth and differentiation^21^. Previous studies of cross-sectional BV and Multiple Sclerosis Severity Score (MSSS) have also reported sub-significant associations with variants in the *PTPRD* region independent from our BV change peak (cross sectional BV top SNP: top SNP: rs1953594, adjusted trend − logP = 4.3; MSSS top SNP: rs10977017, p = 1.02 × 10^–5^)^5,6^. Additionally, variants mapped to *PTPRD* are associated with two sleep disorders that are enriched in PwMS: restless leg syndrome (top SNP rs1836229)^22,23^ and insomnia (top SNP rs10761240)^24,25^. While we did not observe evidence for colocalization between the top BV change association and *PTPRD* expression, it is possible that this study is insufficiently powered to detect colocalization, or that the locus impacts mechanisms that are time-dependent, cell lineage-specific, or independent of *PTPRD* expression.

The top T2LV association is an indel SNP that lies in the regulatory region upstream of *NEDD4L. NEDD4L* (neural precursor cell expressed developmentally down-regulated 4-like) plays a role in axon guidance, neurite growth, synaptic transmission, and pain sensitivity^26,27^. The *NEDD4L* peak showed evidence for colocalization with expression of *NEDD4L* in blood (GTEx). The minor allele of this top SNP (rs11398377*C- deletion) was associated with greater T2LV accumulation and increased gene expression, indicating that downregulation of the gene may have therapeutic potential. Missense SNPs in *NEDD4L* cause abnormal fetal neurodevelopment and brain malformations such as periventricular nodular heterotopia^28^. *NEDD4L* encodes an E3 ubiquitin-protein ligase that regulates several membrane channels and transporters including epithelial (ENaC)^29^ and voltage-gated (NaV)^30^ sodium channels. Both ENaCs and NaVs have been implicated in demyelination and MS pathophysiology, and research is ongoing to investigate the role of sodium channel blockers in preventing axonal damage^31,32^. This link is supported by use of the channel blocker dalfampridine for improvement of walking in PwMS^33^.

While BV and T2LV change traits may be correlated^15^ (eTable 10), they reflect complementary measures of MS progressive biology—BV reduction represents a global measure of brain tissue loss, while T2LV increase reflects immune-mediated injury in the CNS white matter. Both *PTPRD* and *NEDD4L* are strongly expressed in the CNS, particularly in neurons. *PTPRD* is also abundant in oligodendrocytes and oligodendrocyte precursor cells. Oligodendrocytes play an important role in MS because they myelinate neurons. *NEDD4L* is a paralog of *NEDD4*, which promotes ubiquitination and degradation of *RAPGEF2*, one of the few putative MS risk genes with enhanced single cell expression in CNS cells^34,35^.

The IMSGC recently published a large study of MS severity using the age-related MS severity score (ARMSS), and reported association in the *DYSF–ZNF638* locus^4^. Additionally, a smaller study (N = 1813 individuals) examining median longitudinal ARMSS (l-ARMSS) and longitudinal MSSS (l-MSSS) did not find significant association^36^. The IMSGC *DYSF–ZNF638* locus index variant (rs10191329) was not associated with change in BV (beta = − 0.004, p = 0.91) nor change in T2LV in our analysis (beta = 0.07, p = 0.04). This may be due to the greater power in the IMSGC analysis, but it is also possible that the variables we analyzed capture different aspects of the MS disease course. We chose to focus on objective imaging measures rather than clinical measures such as EDSS (on which the ARMSS variable is based) due to certain limitations in these measures. For instance, the EDSS score is numerical and nonlinear (ranging from 0 to 10) and is largely based on mobility, making it a poor proxy for progression.

Similar to the IMSGC MS severity study, we did not find overlap between MS risk loci and our peaks, nor did we observe association with an MS PRS^4^. This suggests that mechanisms driving MS susceptibility may differ from those modulating outcomes influencing disease progression. A prior study in ~ 500 MS patients found that the strongest genetic risk factor for MS (HLA- DRB1*15:01) was associated with reduction in brain parenchymal volume and higher T2 lesion load^37^, however these findings were not replicated in our study.

We acknowledge the following limitations in our study. All but two of our RCTs (ASCEND, ORATORIO) examined RRMS participants, and it is possible that the biological mechanisms underlying MS progression differ between early-stage and late-stage MS^38^. Moreover, the clinical trial participants may not be representative of the general MS population owing to strict inclusion and exclusion criteria used in participant selection of RCTs. Neither peak identified in this study reached genome-wide significance (p < 5 × 10^–8^) or a stricter threshold of p < 2.5 × 10^–8^ to account for multiple testing. Though powered to detect moderate associations with common SNPs, our ability to detect genome-wide associations would be improved with a larger sample size. Additional, well-characterized cohorts are needed for independent replication. Longitudinal MRI data was available for < 2 years, however this reflects a small proportion of the average MS patient’s disease course, and longer follow-up would provide more accurate outcomes on BV and T2LV changes. Several of the trials had additional longitudinal data available from open label extension studies, however including these data would have introduced complexities such as variable lengths of follow-up, and differential handling of placebo and treatment arms when accounting for pseudoatrophy at treatment initiation. We therefore limited our analyses to data from the placebo-controlled period. While we endeavored to account for pseudoatrophy by re-baselining at 24 weeks, it is possible this did not fully capture all pseudoatrophy. We chose to re-baseline using week 24 measures to have a reasonable duration of MRI follow-up for GWAS.

This work highlights the utility of using clinical trial data for genetic analyses. RCTs have assigned treatment arms and systematic imaging collection, and genetic analyses of RCTs are uniquely positioned to leverage objective, quantitative measurements to advance our understanding of MS disease course. Furthermore, the MRI analysis methods used to quantify atrophy and T2LV were standardized within studies, and to the extent possible, across the studies used in the meta-analysis, because all studies utilized the same centralized MRI reading center (NeuroRx). Finally, our findings complement existing risk studies by identifying additional genetic factors related to different aspects of the disease.

In conclusion, this study identified two novel putative loci (*PTPRD*, *NEDD4L*) that may impact MS disease progression. Investigation in additional cohorts are warranted to validate these findings.

### Supplementary Information

Supplementary Information 1.

Supplementary Tables.

## Data Availability

Individual level data is not available due to the use of clinical trial data. Summary statistics may be available upon request. Requests may be sent to Stephanie Loomis, stephanie.loomis@biogen.com.
