# Supplementary material for: Genome-wide study of longitudinal brain imaging measures of multiple sclerosis progression across six clinical trials
Source: Sci Rep. 2023 Aug 31;13:14313. doi: 10.1038/s41598-023-41099-0 (PMC10471679; doi:10.1038/s41598-023-41099-0)
Supplement: Supplementary file 1 — Supplementary Information 1. [file 41598_2023_41099_MOESM1_ESM.docx]

**eFigure 1. Distributions of annualized Brain Volume (BV) change for each trial: (A) untransformed and (B) rank-based inverse normal transformed**

1. Untransformed annualized BV change


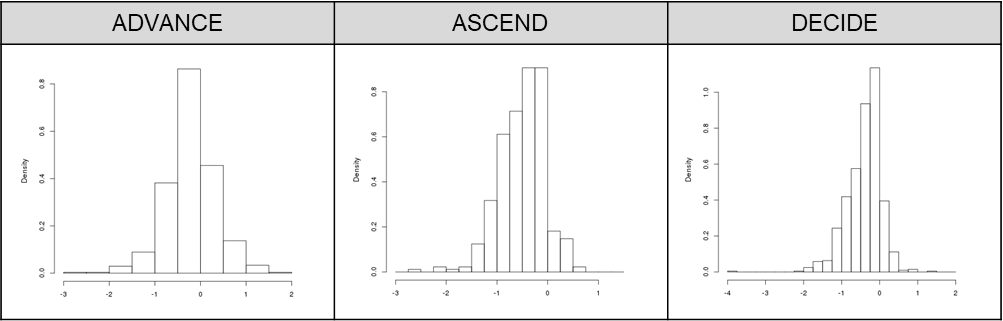


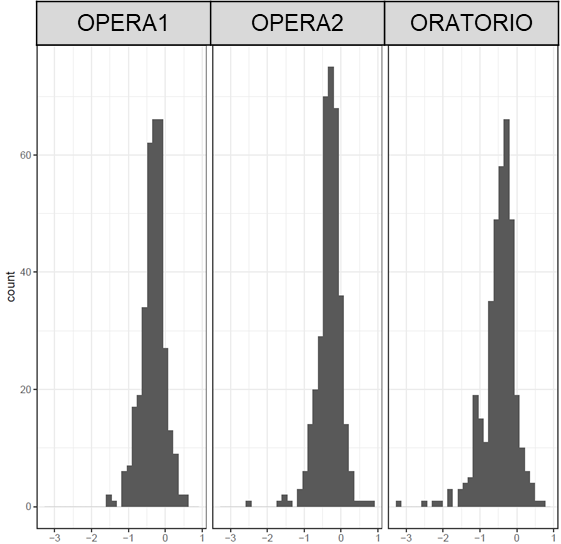


1. Rank-based inverse normal transformed annualized BV change


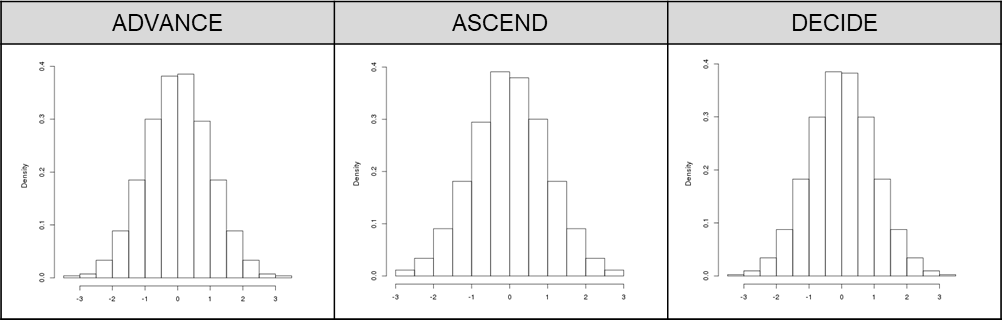


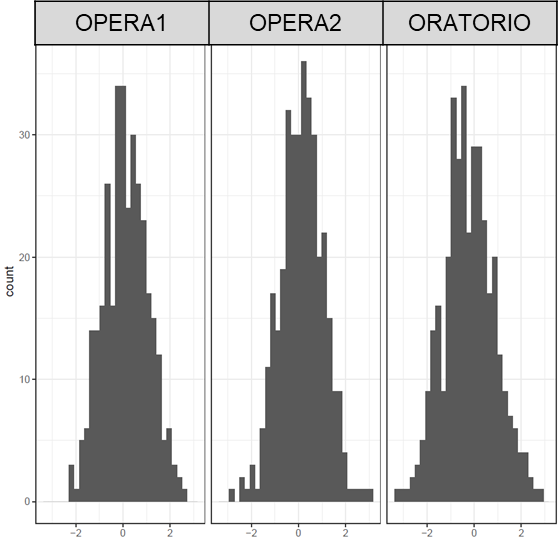


**Abbreviations**: BV, brain volume; T2LV, T2 lesion volume.

**eFigure 2. Distributions of annualized T2 Lesion Volume (T2LV) change for each trial: (A) untransformed and (B) rank-based inverse normal transformed**

1. Untransformed annualized T2LV change


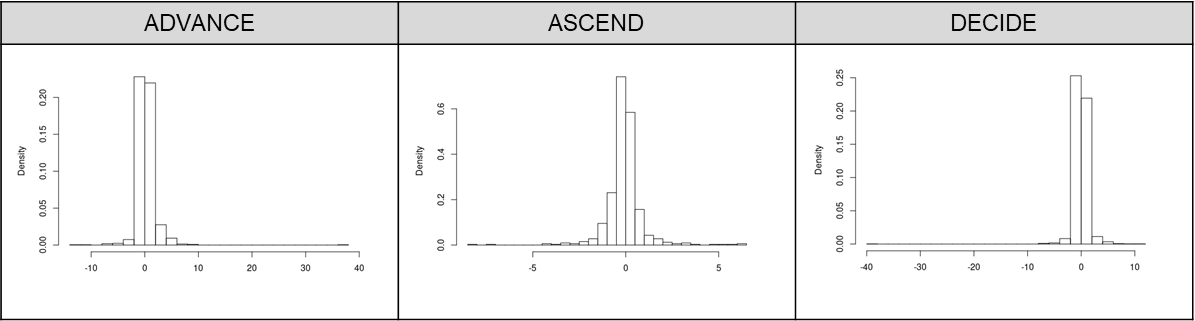


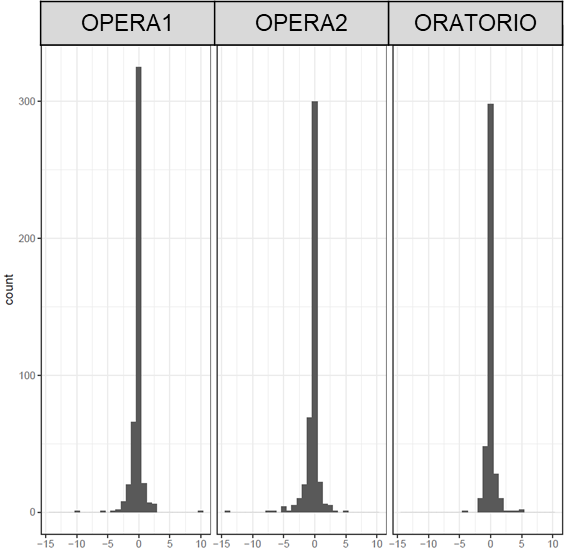


1. Rank-based inverse normal transformed annualized T2LV change

**
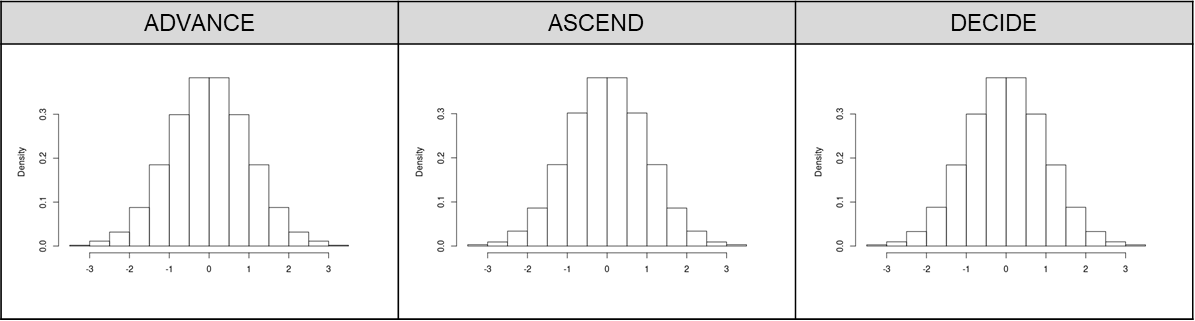
**

**
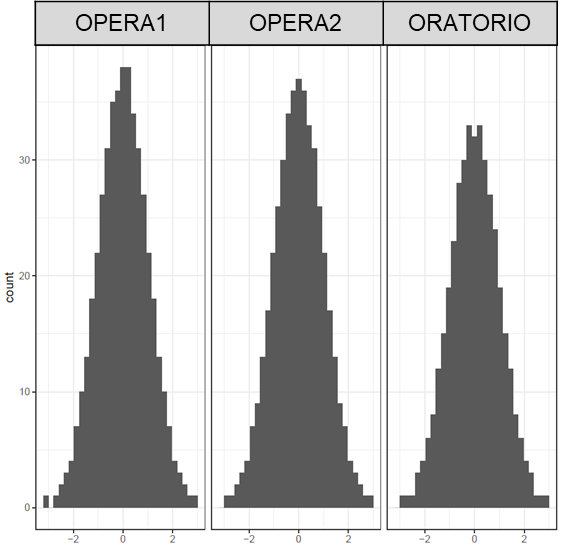
**

**Abbreviations**: BV, brain volume; T2LV, T2 lesion volume.

**eFigure 3. Quantile-quantile (QQ) plots from GWAS meta-analysis: (A) Brain Volume (BV) change; (B) T2 Lesion Volume (T2LV) change**

1. BV change (λ = 1.008)


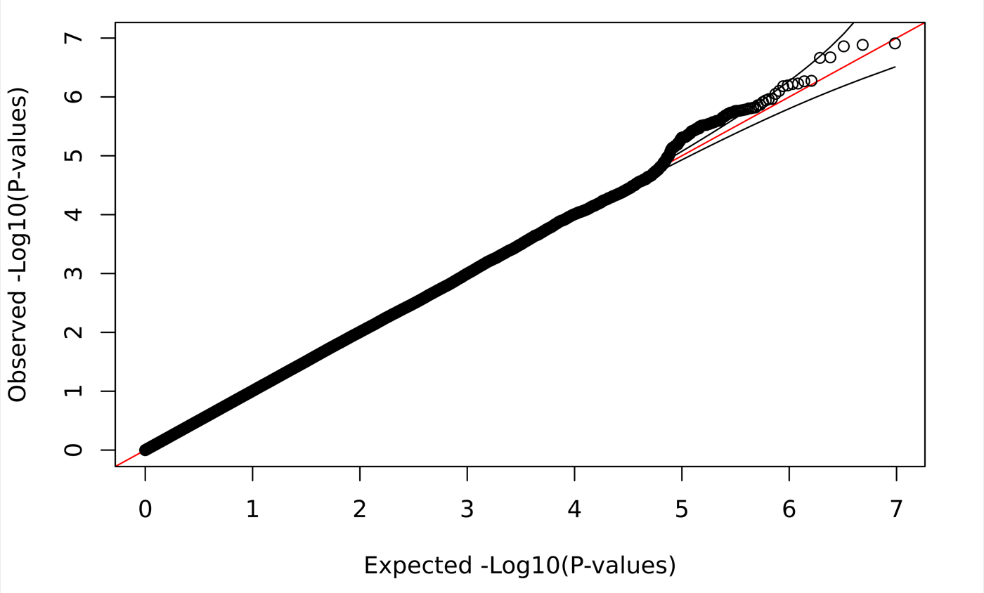


1. T2LV change (λ = 1.019)


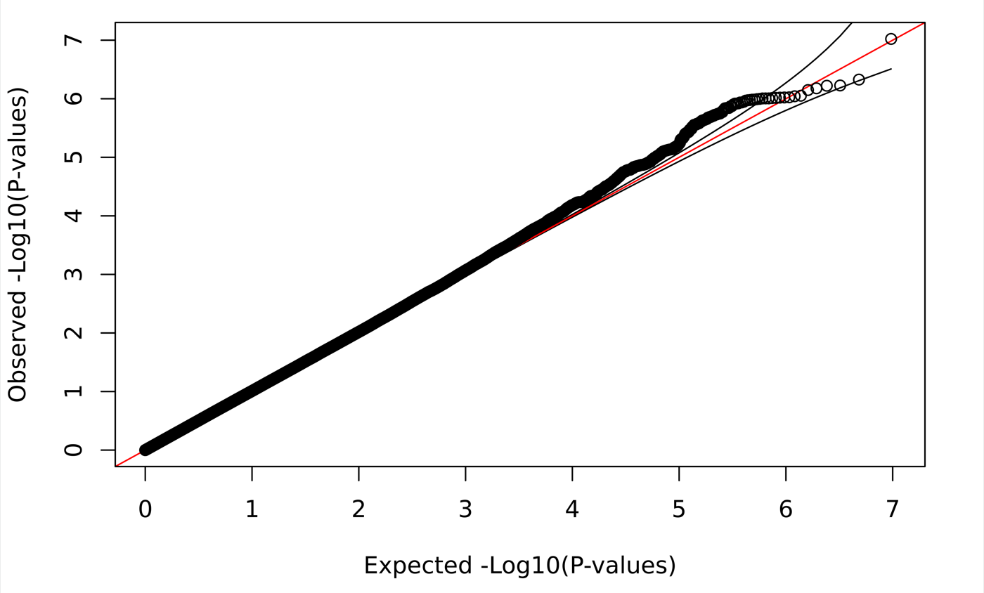


**Abbreviations**: BV, brain volume; GWAS, genome-wide association study; T2LV, T2 lesion volume.

**eTable 1. Characteristics of the three Biogen randomized controlled trials in our GWAS**

| **Trial** | **ADVANCE** | **ASCEND** | **DECIDE** |
| --- | --- | --- | --- |
| **Active arm** | Pegylated IFNβ (subcutaneous) | Natalizumab | Daclizumab |
| **Control arm** | Placebo | Placebo | IFNβ (intramuscular) |
| **MS diagnosis** | RRMS | SPMS | RRMS |
| **National Clinical Trial (NCT) Identifier** | 00906399 | 01416181 | 01064401 |
| **Trial dates** | 2009-2013 | 2011-2016 | 2010-2014 |
| **Inclusion Criteria** | - RMS diagnosis - EDSS 0-5 - 2+ clinically documented relapses over the past 3 years (with at least one relapse in past year) | - SPMS diagnosis - EDSS 3-6.5 - MSSS ≥4 - Documented disease progression over past year | - RMS diagnosis - EDSS 0-5 - Use of contraception during and 4 months after last dose |
| **Exclusion Criteria** | - Major chronic disease - Pregnancy - Nursing - Treatment with IFN β >4 weeks and/or within 6 months preceding baseline | - 1+ clinical relapse in previous 3 months - T25FW test of >30 seconds during screening period - Contraindications for MRI - History of certain chronic/infectious disease, treatment with other MS medications, immunosuppressant use | - MS relapse within 50 days prior to randomization - Previous treatment with Daclizumab HYP - Known hypersensitivity to study drugs or their excipients - History of certain chronic/infectious diseases, substance abuse, seizures, neurological instability |

**Abbreviations**: EDSS, expanded disability status score; MSSS, multiple sclerosis severity score; RRMS, relapsing remitting multiple sclerosis; SPMS, secondary progressive multiple sclerosis; T25FW, timed 25-foot walk.

**eTable 2. Characteristics of the three Roche/Genentech randomized controlled trials in our GWAS**

| **Trial** | **OPERA1** | **OPERA2** | **ORATORIO** |
| --- | --- | --- | --- |
| **Active arm** | Ocrelizumab | Ocrelizumab | Ocrelizumab |
| **Control arm** | IFNβ (subcutaneous) | IFNβ (subcutaneous) | Placebo |
| **MS diagnosis** | RRMS | RRMS | PPMS |
| **National Clinical Trial (NCT) ID** | 01247324 | 01412333 | 01194570 |
| **Trial dates** | 2011-2015 | 2011-2015 | 2011-2015 |
| **Inclusion Criteria** | - Diagnosis of MS - EDSS 0-5.5 - 2+ documented clinical attacks within last 2 years or 1 clinical attack in previous years (not within past 30 days) - Neurologic stability for past 30 days | - Diagnosis of MS - EDSS 0-5.5 - 2+ documented clinical attacks within last 2 years or 1 clinical attack in previous years (not within past 30 days) - Neurologic stability for past 30 days | - PPMS diagnosis - EDSS 3-6.5 - Disease duration < 15 years if EDSS >5.0; <10 years if EDSS ≥5.0 - Use of contraception during and 44 weeks after last dose |
| **Exclusion Criteria** | - PPMS diagnosis - Disease duration >10 years in patients with EDSS$\leq$2 - Contraindications for MRI - Pregnancy or lactation - History of other neurological disorders, certain chronic/infectious diseases, corticosteroid or immunosuppressant use | - PPMS diagnosis - Disease duration >10 years in patients with EDSS$\leq$2 - Contraindications for MRI - Pregnancy or lactation - History of other neurological disorders, certain chronic/infectious diseases, corticosteroid or immunosuppressant use | - RRMS, SPMS, PRMS - Contraindications for MRI - History of other neurological disorders, certain chronic/infectious diseases, corticosteroid or immunosuppressant use |

**Abbreviations**: EDSS, expanded disability status score; RRMS, relapsing remitting multiple sclerosis; PPMS, primary progressive multiple sclerosis; PRMS, progressive relapsing multiple sclerosis; T25FW, timed 25-foot walk.

**eTable 3. SNPs and samples remaining after exclusions at each QC step for Biogen trials (ADVANCE, ASCEND and DECIDE).**

|  | Genotyping batch 1  2017; ASCEND and DECIDE | | Genotyping batch 2  2018; ADVANCE | |
| --- | --- | --- | --- | --- |
| QC Step | **N Samples** | **N SNPs** | **N Samples** | **N SNPs** |
| Genotyped data | 3,053 | 830,115 | 1,037 | 805,467 |
| Remove genotyping controls | 3,021 | 830,115 | 969 | 805,467 |
| Exclude variants with missingness >1% | 3,021 | 761,435 | 969 | 755,133 |
| Exclude low frequency variants (MAF<0.01) | 3,021 | 617,223 | 969 | 614,280 |
| Exclude variants out of HWE >10-50 | 3,021 | 616,851 | 969 | 6142,33 |
| Exclude samples with missingness > 2% | 2,988 | 616,851 | 961 | 614,233 |
| Sex check | 2,983 | 616,851 | 950 | 614,233 |
| Check for samples with excess heterozygosity | 2,972 | 616,851 | 942 | 614,233 |
| Exclude related individuals (IBD analysis) | 2,952 | 616,851 | 941 | 614,233 |
| Imputed variants |  | 48,935,182 |  | 48,935,198 |
| Exclude variants with Rsq<0.3 |  | 19,068,517 |  | 17,565,382 |
| Include only samples from trials used in this analysis | ASCEND: 590  DECIDE: 1,113 |  | ADVANCE: 746 |  |
| Exclude PCA outliers | ASCEND: 582  DECIDE: 1,076 |  | ADVANCE: 690 |  |
| Exclude variants with MAF>0.01 and HWE>10-50 (in each GWAS) and samples with phenotype – BV change; T2LV change | ASCEND: 353; 435  DECIDE: 821; 886 | ASCEND: 9,315,378  DECIDE: 9,408,057 | ADVANCE: 540; 505 | ADVANCE: 9,500,007 |

**eTable 4. Peak SNPs from meta-analysis of Brain Volume (BV) change GWAS by trial**

|  | | | | ADVANCE  (N = 540) | | ASCEND  (N = 353) | | DECIDE  (N = 821) | | OPERA 1  (N = 581) | | OPERA 2  (N = 577) | | ORATORIO  (N = 529) | | Meta-analysis^a^  (Total N = 3,401) | |
| --- | --- | --- | --- | --- | --- | --- | --- | --- | --- | --- | --- | --- | --- | --- | --- | --- | --- |
| **SNP** | **CHR** | **BP** | **A1/A2** | **Beta (SE)** | **P-value** | **Beta (SE)** | **P-value** | **Beta (SE)** | **P-value** | **Beta (SE)** | **P-value** | **Beta (SE)** | **P-value** | **Beta (SE)** | **P-value** | **Beta (SE)** | **P-value** |
| rs10491610 | 9 | 9537211 | T/G | -0.17 (0.08) | 0.038 | -0.14 (0.1) | 0.152 | -0.14 (0.07) | 0.043 | -0.2 (0.1) | 0.051 | -0.21 (0.09) | 0.024 | -0.26 (0.1) | 0.009 | -0.18 (0.04) | 5.43x10^-7^ |
| rs17772815 | 9 | 9540766 | C/G | -0.18 (0.08) | 0.033 | -0.15 (0.1) | 0.141 | -0.15 (0.07) | 0.033 | -0.16 (0.1) | 0.111 | -0.22 (0.09) | 0.018 | -0.25 (0.1) | 0.013 | -0.18 (0.04) | 6.57x10^-7^ |
| rs77321193 | 9 | 9547291 | C/A | -0.18 (0.08) | 0.031 | -0.15 (0.1) | 0.138 | -0.15 (0.07) | 0.027 | -0.16 (0.1) | 0.111 | -0.22 (0.09) | 0.018 | -0.25 (0.1) | 0.014 | -0.18 (0.04) | 5.33x10^-7^ |
| rs736043 | 9 | 9553360 | C/T | -0.18 (0.08) | 0.032 | -0.15 (0.1) | 0.138 | -0.15 (0.07) | 0.023 | -0.16 (0.1) | 0.104 | -0.21 (0.09) | 0.021 | -0.23 (0.1) | 0.02 | -0.18 (0.04) | 6.39x10^-7^ |
| rs76647005 | 9 | 9558543 | C/T | -0.18 (0.08) | 0.035 | -0.15 (0.1) | 0.128 | -0.16 (0.07) | 0.023 | -0.16 (0.1) | 0.111 | -0.22 (0.09) | 0.015 | -0.23 (0.1) | 0.025 | -0.18 (0.04) | 5.93x10^-7^ |
| rs77127788 | 9 | 9561124 | A/G | -0.17 (0.08) | 0.037 | -0.16 (0.1) | 0.11 | -0.15 (0.07) | 0.025 | -0.16 (0.1) | 0.11 | -0.23 (0.09) | 0.014 | -0.22 (0.1) | 0.027 | -0.18 (0.04) | 6.04x10^-7^ |
| rs6477402 | 9 | 9562254 | C/T | -0.17 (0.08) | 0.043 | -0.16 (0.1) | 0.11 | -0.16 (0.07) | 0.022 | -0.14 (0.1) | 0.148 | -0.21 (0.09) | 0.025 | -0.24 (0.1) | 0.017 | -0.18 (0.04) | 8.89x10^-7^ |
| rs137996531 | 10 | 71059147 | A/G | 0.26 (0.26) | 0.309 | 0.06 (0.23) | 0.794 | 0.72 (0.2) | 0.0004 | 0.14 (0.24) | 0.567 | 0.72 (0.2) | 0.0003 | 0.67 (0.27) | 0.014 | 0.46 (0.09) | 7.90x10^-7^ |

**Abbreviations**: A1, Allele 1 (effect allele); A2, Allele 2; BP, genomic position in base pairs; CHR, chromosome; GWAS, genome-wide association study; P, P-value; SNP, single nucleotide polymorphism.

**^a^** Fixed-effect meta-analysis results with p < 1x10^-6^ and no significant heterogeneity (Cochrane’s Q p-value > 0.05 for all SNPs).

- indicates missing results for a trial.

(note: eTable 5 can be found in the Supplementary 2 excel file)

**eTable 6. Peak SNPs from meta-analysis of change in T2 Lesion Volume (T2LV) GWAS studies, by clinical trial**

|  | | | | ADVANCE  (N = 505) | | ASCEND  (N = 435) | | DECIDE  (N = 886) | | OPERA 1  (N = 581) | | OPERA 2  (N = 577) | | ORATORIO  (N = 529) | | Meta-analysis^a^  (Total N = 3,513) | |
| --- | --- | --- | --- | --- | --- | --- | --- | --- | --- | --- | --- | --- | --- | --- | --- | --- | --- |
| **SNP** | **CHR** | **BP** | **A1/A2** | **Beta (SE)** | **P-value** | **Beta (SE)** | **P-value** | **Beta (SE)** | **P-value** | **Beta (SE)** | **P-value** | **Beta (SE)** | **P-value** | **Beta (SE)** | **P-value** | **Beta (SE)** | **P-value** |
| rs11398377 | 18 | 55710518 | GC/G- | -0.19 (0.09) | 0.03 | -0.16 (0.08) | 0.048 | -0.05 (0.06) | 0.399 | -0.13 (0.09) | 0.126 | -0.35 (0.09) | 0.0001 | -0.32 (0.09) | 0.001 | -0.18 (0.03) | 9.52x10^-8^ |
| rs9955426 | 18 | 55714622 | C/T | -0.17 (0.09) | 0.064 | -0.16 (0.08) | 0.051 | -0.05 (0.06) | 0.397 | -0.12 (0.09) | 0.184 | -0.35 (0.09) | 0.0001 | -0.29 (0.09) | 0.002 | -0.17 (0.03) | 6.00x10^-7^ |
| rs8093023 | 18 | 55720092 | A/G | -0.17 (0.09) | 0.069 | -0.16 (0.08) | 0.055 | -0.05 (0.06) | 0.421 | -0.12 (0.09) | 0.184 | -0.35 (0.09) | 0.0001 | -0.29 (0.09) | 0.002 | -0.17 (0.03) | 5.94x10^-7^ |
| rs8097163 | 18 | 55720610 | A/G | -0.17 (0.09) | 0.07 | -0.16 (0.08) | 0.055 | -0.05 (0.06) | 0.423 | -0.12 (0.09) | 0.184 | -0.35 (0.09) | 0.0001 | -0.29 (0.09) | 0.002 | -0.17 (0.03) | 7.11x10^-7^ |
| rs6566928 | 18 | 55725195 | C/T | -0.16 (0.09) | 0.089 | -0.16 (0.08) | 0.059 | -0.05 (0.06) | 0.432 | -0.12 (0.09) | 0.178 | -0.35 (0.09) | 0.0001 | -0.29 (0.09) | 0.002 | -0.17 (0.03) | 9.07x10^-7^ |
| rs6566931 | 18 | 55725783 | A/G | -0.15 (0.09) | 0.093 | -0.16 (0.08) | 0.059 | -0.05 (0.06) | 0.437 | -0.12 (0.09) | 0.178 | -0.35 (0.09) | 0.0001 | -0.29 (0.09) | 0.002 | -0.17 (0.03) | 9.51x10^-7^ |
| rs5005280 | 18 | 55725820 | G/A | -0.15 (0.09) | 0.093 | -0.16 (0.08) | 0.059 | -0.05 (0.06) | 0.436 | -0.12 (0.09) | 0.178 | -0.35 (0.09) | 0.0001 | -0.29 (0.09) | 0.002 | -0.17 (0.03) | 9.45x10^-7^ |
| rs1942562 | 18 | 55725858 | C/T | -0.15 (0.09) | 0.093 | -0.16 (0.08) | 0.059 | -0.05 (0.06) | 0.438 | -0.12 (0.09) | 0.178 | -0.35 (0.09) | 0.0001 | -0.29 (0.09) | 0.002 | -0.17 (0.03) | 9.57x10^-7^ |
| rs4371241 | 18 | 55725925 | A/C | -0.15 (0.09) | 0.093 | -0.16 (0.08) | 0.059 | -0.05 (0.06) | 0.44 | -0.12 (0.09) | 0.178 | -0.35 (0.09) | 0.0001 | -0.29 (0.09) | 0.002 | -0.17 (0.03) | 9.68x10^-7^ |
| rs1942563 | 18 | 55725956 | C/G | -0.15 (0.09) | 0.094 | -0.16 (0.08) | 0.059 | -0.05 (0.06) | 0.441 | -0.12 (0.09) | 0.178 | -0.35 (0.09) | 0.0001 | -0.29 (0.09) | 0.002 | -0.17 (0.03) | 9.73x10^-7^ |
| rs4461163 | 18 | 55726087 | T/G | -0.16 (0.09) | 0.089 | -0.16 (0.08) | 0.05 | -0.05 (0.06) | 0.457 | -0.13 (0.09) | 0.156 | -0.35 (0.09) | 0.0001 | -0.3 (0.09) | 0.002 | -0.17 (0.03) | 6.64x10^-7^ |
| rs1942564 | 18 | 55726105 | A/G | -0.15 (0.09) | 0.094 | -0.16 (0.08) | 0.059 | -0.05 (0.06) | 0.444 | -0.12 (0.09) | 0.178 | -0.35 (0.09) | 0.0001 | -0.29 (0.09) | 0.002 | -0.17 (0.03) | 9.87x10^-7^ |
| rs1942565 | 18 | 55726152 | C/T | -0.15 (0.09) | 0.094 | -0.16 (0.08) | 0.059 | -0.05 (0.06) | 0.444 | -0.12 (0.09) | 0.178 | -0.35 (0.09) | 0.0001 | -0.29 (0.09) | 0.002 | -0.17 (0.03) | 9.88x10^-7^ |
| rs4375742 | 18 | 55726189 | T/C | -0.15 (0.09) | 0.096 | -0.16 (0.08) | 0.056 | -0.05 (0.06) | 0.447 | -0.12 (0.09) | 0.178 | -0.34 (0.09) | 0.0001 | -0.29 (0.09) | 0.002 | -0.17 (0.03) | 9.86x10^-7^ |
| rs12237424 | 9 | 2442058 | A/G | -0.17 (0.12) | 0.179 | -0.15 (0.13) | 0.224 | -0.42 (0.1) | 1x10^-5^ | -0.09 (0.11) | 0.385 | -0.23 (0.11) | 0.037 | -0.26 (0.13) | 0.048 | -0.23 (0.05) | 4.71x10^-7^ |
| rs145565113 | 5 | 176921677 | A/G | -0.51 (0.32) | 0.11 | -0.8 (0.29) | 0.006 | -0.54 (0.23) | 0.018 | -0.76 (0.22) | 0.001 | -0.18 (0.22) | 0.411 | -0.26 (0.23) | 0.26 | -0.49 (0.10) | 8.88x10^-7^ |

**Abbreviations**: A1, Allele 1 (effect allele); A2, Allele 2; BP, genomic position in base pairs; CHR, chromosome; GWAS, genome-wide association study; P, P-value; SNP, single nucleotide polymorphism.

**^a^** Fixed-effect meta-analysis results with p < 1x10^-6^ and no significant heterogeneity (Cochrane’s Q p-value > 0.05 for all SNPs).

**eTable 10. Correlation between annualized changes in Brain Volume (BV) and T2 Lesion Volume (T2LV)**

|  |  | **GWAS Sample Size** | |  |  |
| --- | --- | --- | --- | --- | --- |
| **Trial ^a^** | **MS diagnosis** | **BV Change**  **(Total N = 3,401)** | **T2LV Change**  **(Total N = 3,513)** | **Correlation Coefficient** | **P** |
| ADVANCE | RRMS | 540 | 505 | -0.07 | 0.12 |
| ASCEND | SPMS | 353 | 435 | -0.05 | 0.35 |
| DECIDE | RRMS | 821 | 886 | -0.11 | 1.57x10^-3^ |
| OPERA 1 | RRMS | 581 | 581 | -0.02 | 0.73 |
| OPERA 2 | RRMS | 577 | 577 | 0.001 | 0.99 |
| ORATORIO | PPMS | 529 | 529 | -0.32 | 1.17x10^-9^ |

**Abbreviations**: MS, multiple sclerosis; P, P-value; PPMS, primary progressive MS ; RRMS, relapsing remitting MS ; SPMS, secondary progressive MS .

**^a^** Randomized Controlled Trial

**^b^** Pearson Correlation Coefficient
